# Evaluation of a Computerized Decision Support Intervention to Decrease Use of Anti-Pseudomonal Carbapenems in Penicillin Allergic Patients

**DOI:** 10.3390/antibiotics5010007

**Published:** 2016-01-15

**Authors:** Christina Caplinger, Garret Smith, Richard Remington, Karl Madaras-Kelly

**Affiliations:** 1 Infectious Diseases Research Fellow, Pharmacy Service, Boise Veterans Affairs Medical Center, Bldg T111, 500 W Fort St. Boise, ID 83702, USA; Christina.caplinger@va.gov; 2 Pharmacy Informatics Program Manager, Pharmacy Service, Boise Veterans Affairs Medical Center, 500 W Fort St. Boise, ID 83702, USA; Garret.smith@va.gov; 3 Biostatistician, Research Service, Boise Veterans Affairs Medical Center and Quantified Inc. T111, 500 W Fort St. Boise, ID 83702, USA; Remington@quantified.us; 4 Clinical Pharmacist, Pharmacy Service, Boise Veterans Affairs Medical Center, and Professor of Pharmacy, College of Pharmacy, Idaho State University, Bldg T111, 500 W Fort St. Boise, ID 83702, USA

**Keywords:** antimicrobial stewardship, computerized decision support system, antipseudomonal, carbapenem, β-lactam allergy, β-lactam cross-reactivity

## Abstract

Allergies to β-lactam antibiotics are commonly documented in hospitalized patients; however, true allergy is uncommon. Cross-reactivity rates for advanced generation cephalosporins and carbapenems are low; particularly for patients without a history of symptoms consistent with type 1 hypersensitivity. We observed that providers preferentially prescribed antipseudomonal carbapenems (APC) over advanced generation cephalosporins for patients with β-lactam allergy history, including those with low risk for antimicrobial-resistant infections. Information was inserted into the computerized decision support system (CDSS) to aid clinicians in assessing β-lactam cross-reactivity risk and selecting appropriate therapy. A retrospective evaluation was conducted in a small hospital to assess the impact of the CDSS changes in APC prescribing. Inpatients (*n* = 68) who received at least one APC dose during hospitalization over a 13 month pre-intervention period were compared to inpatients who received an APC during the 15 month post-intervention period (*n* = 59) for documented APC indications and β-lactam allergy history. APC initiations were measured and corrected per 1000 patient-days; interrupted time-series analysis was performed to assess changes in use before and after implementation. Aggregate monthly APC initiations decreased from 7.01 to 6.14 per 1000 patient-days after the implementation (*p* = 0.03). Post-intervention APC initiations for patients with low-risk β-lactam histories decreased from 92% to 83% (*p* = 0.17). No adverse events were observed in patients with low-risk β-lactam histories. The intervention was associated with a reduction in APC initiations.

## 1. Introduction

Carbapenems are broad-spectrum β-lactam antibiotics commonly reserved for serious gram-negative infections, including those caused by multiple drug-resistant (MDR) *Pseudomonas aeruginosa* and extended-spectrum β-lactamase (ESBL) producing organisms [1,2]. Carbapenem use has increased in the United States as well as in the Veterans Affairs (VA) Healthcare System [3]. In recent years, carbapenem-resistant *Enterobacteriaceae* (CRE), including pan-resistant *Klebsiella pneumoniae* carbapenemase (KPC), have emerged in the United States and around the world, and many infections associated with these organisms are both costly and lethal due to unavailability of antimicrobial agents with activity against them [4,5,6,7]. Antimicrobial stewardship is recommended as one of eight core measures for limiting the spread of CRE by the Centers for Disease Control (CDC) [7,8]. In particular, the CDC recommends limiting unnecessary exposure to broad-spectrum antimicrobials which include antipseudomonal carbapenems (APC). Computerized Decision Support (CDSS)-based interventions are effective methods of stewardship that can be directed at clinicians at the time of antimicrobial selection; thus enhancing efforts to curtail unnecessary APC use before antimicrobial stewards perform audit and feedback [9]. We implemented a CDSS approach to direct selection of APC therapy at the time of antimicrobial selection and order entry.

This evaluation was conducted at a small VA teaching hospital with a 10-bed intensive care unit, a 46-bed combined medical/surgical unit, and a 32-bed Community Living Center.

It was observed that much APC use in this facility appeared to be in patients with a history of penicillin allergy documented in the electronic medical record. Penicillin allergies are noted in up to 10% of all inpatients treated with antimicrobials and of those, about 10% demonstrate an allergic response when skin testing is performed [10]. Many patients with a history of penicillin allergy have a low risk of cross-reactivity with other β-lactam antibiotics as their allergies are not associated with IgE-mediated symptoms (e.g., urticaria, hives, or anaphylaxis) [11,12]. Penicillin skin tests may be utilized to verify allergy status, however, practice parameters and product labelling recommend that only well-trained personnel perform testing, or that only specialty allergy practitioners are to interpret these tests in the case of indeterminate results [10,11,13,14]. Many small facilities, including ours, do not have an allergist available to train clinicians on performance of the test or to evaluate indeterminate skin test findings, making it difficult to determine existence of penicillin allergy with this methodology. Penicillin cross-reactivity in patients with allergy verified by penicillin skin testing is reported in literature to be 2%–3% for cephalosporins and 1% or less for carbapenems [15,16] UpToDate is a source of evidence-based medical diagnostic and treatment information that is available to practitioners in many healthcare systems, including the VA [17,18]. A recent survey of graduates from three medical schools indicated that UpToDate was most frequently used resource used for learning about antimicrobial prescribing with more than 90% of respondents indicating use of this reference [19]. UpToDate recommends that if allergy skin testing is not readily available that clinicians stratify patient risk of cross-reactivity based upon the duration of time passed since the potential adverse reaction occurred and the features of prior penicillin reaction, then consider prescribing a cephalosporin to patients deemed at “low risk” for cross-reactivity [11,14,20,21].

Based on our observations of medical resident antimicrobial prescribing patterns we speculated that clinicians would frequently forgo the risk stratification strategy for patients with penicillin allergy and select an APC based on the perceived lower risk of cross-reactivity, irrespective of the infectious indication for APCs relative to cephalosporins.

The purpose of this pre/post intervention medication utilization evaluation (MUE) was to investigate the impact of implementing an electronic ordering prompt that directed providers to risk-stratify penicillin cross-reactivity potential and assess indications for appropriate APC therapy prior to ordering antimicrobials.

## 2. Results and Discussion

### 2.1. Results

There were 127 APC initiations over the evaluative period: 68 patients received an APC during the 13 month pre-implementation period and 59 patients received an APC during the 15 month post-implementation period. Patients were primarily elderly, male, and were most frequently admitted to the general medicine/surgical ward. Overall, 56.7% of patients had documentation of prior β-lactam allergy, of which 93.0% had documentation of low risk β-lactam allergy. There were no significant differences in patient characteristics or indications for APC use pre and post intervention (Table 1).

**Table 1:** Patient Demographics and Indications for Antipseudomonal Carbapenem (APC) Use.

Demographics and Indications	Pre-Implementation (*n* = 68)	Post-Implementation (*n* = 59)	*p*
**Patient Demographics**			
Age (years), Mean (SD)	71.4 (14.3)	69.5 (12.4)	0.43
Male sex, *n* (%)	66 (97.1%)	58 (98.3%)	0.64
Non-ICU admission, *n* (%)	41 (60.3%)	42 (71.2%)	0.19
Duration of APC therapy, Mean (SD)	4.4 (4.07)	4.0 (2.60)	0.52
**APC Initiations by Indication, *n* (%)**			
History of any β-lactam allergy	41 (60.3%)	32 (54.2%)	0.59
β-lactam allergy (high-risk)	3 (7.3%)	6 (18.7%)	0.17
β-lactam allergy (low-risk)	38 (92.7%)	26 (81.3%)	0.17
β-lactam use within past 90 days	15 (22.1%)	14 (23.7%)	0.99
Severe infection, pancreatitis, and/or sepsis	19 (27.9%)	24 (40.7%)	0.19
Suspected/confirmed MDRO ^†^	7 (10.3%)	5 (8.5%)	0.96
>1 clinical indication noted	22 (32.4%)	30 (50.8%)	0.05
No indication specified	7 (10.3%)	5 (8.5%)	0.96

^†^ MDRO: Multiple Drug-Resistant Organism (includes extended-spectrum β-lactamase producing organism or multiple drug resistant *Pseudomonas aeruginosa*).

**Figure 1:**
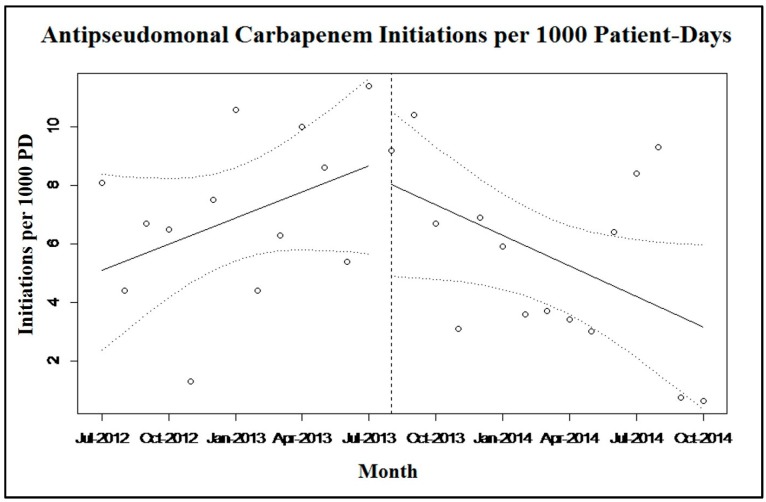
Aggregated monthly APC initiations per 1000 patient-days, before and after the electronic intervention was implemented (indicated by vertical dashed line and 95% confidence intervals indicated by sloped dashed lines).

The average monthly rate of APC initiations was increasing prior to implementation of the antimicrobial menu changes, as indicated by a positive slope of the regression line (slope = 0.27 initiations/1000 PD; *p* = 0.20) (Figure 1). A negative post-implementation slope indicated a decrease in average monthly APC initiations (slope = −0.33 initiations/1000 PD; *p* = 0.06). The change in slope post-intervention was negative at −0.60 (95% CI-1.12, −0.08; *p* = 0.03), indicating that the APC ordering menu intervention was associated with the reduction in APC initiation.

APC initiations for patients who had a documented allergy to β-lactam antibiotics declined minimally post-intervention from 60.3% to 54.2% (*p* = 0.59). The percentage of patients with a documented low-risk history for β-lactam cross reactivity declined from 55.9% before the intervention to 44.1% after the intervention (*p* = 0.21).

There were no documented adverse events for patients with a history of β-lactam allergy who were administered an APC. Additionally, there were 46 patients who received cefepime during the post-intervention phase of which 12 had a β-lactam allergy. Of these, 1 patient experienced an adverse event possibly related to cefepime administration. This patient developed a skin rash, erythema, and bullae formation, but also received concomitant vancomycin and ciprofloxacin. It is unknown if cefepime was the primary agent that precipitated the adverse reaction. The patient’s prior allergy history was documented as a “skin rash/fever” that occurred five years prior to administration of cefepime, with no indication of urticaria, anaphylaxis, or other IgE-mediated symptoms. Therefore; treatment with a cephalosporin was consistent with the CPRS antimicrobial ordering menu recommendations. This patient’s condition improved after stopping the aforementioned antibiotics, and received a course of levofloxacin during the remainder of his hospital stay without issue.

### 2.2. Discussion

APC use was increasing prior to implementing the CPRS antimicrobial ordering menu changes at our facility. After implementing the menu changes which provided guidance on appropriate APC use, especially in patients with history of β-lactam allergy, the number of APC initiations decreased, and the change in slope post-intervention indicated that the reduction in APC initiations was associated with implementation of the menu changes. Other important findings include the observation that more than 50% of APC initiations were prescribed for patients with a documented history of β-lactam allergy and more than 90% of these histories indicated a low risk for β-lactam cross-reactivity. Many of these patients could potentially have been treated with a cephalosporin with narrower spectrum of coverage. It is important to recognize that our intent was not to curtail all APC use, but only use in patients with both a low risk for penicillin cross-reactivity and weak indications for APC coverage. It is also important to recognize that the concept of carbapenem cross-reactivity in patients with a history of β-lactam allergy is controversial. Early studies suggested that cross-reactivity with skin testing was as high as 50%; however, more recent skin testing and clinical administration studies suggest cross reactivity rates of <1% which may have lead clinicians to classify most patients with a history of β-lactam allergy as candidates for APC therapy [22]. Of note, 10.5% of patients in our analysis still received an APC despite a history of high risk for β-lactam cross reactivity suggesting a risk/benefit assessment favoring APC coverage over the potential limited concern for cross-reactivity with an APC. In select cases this may have been appropriate as the use of aztreonam can be associated with β-lactamase induction and poor outcome with monotherapy [23]. Adverse event rates were low and no adverse events were identified in patients with a β-lactam allergy who received an APC or an advanced generation cephalosporin prescribed in accordance with the recommendations embedded in the CPRS antimicrobial ordering menus.

The findings are important because it appears that some patients were receiving APCs based solely on their allergy history, regardless of the actual infectious indication for APC therapy. Moreover, the majority of patients with a β-lactam allergy who received an APC were at low-risk for cross-reactivity with other β-lactams such as advanced generation cephalosporins. The majority of hospitals in the U.S. are less than 100 beds, and many smaller facilities do not have ready access to an allergist to train clinical personnel to perform skin testing or to interpret indeterminate results that may be encountered when performing penicillin skin testing [24]. In addition, exposure to APCs are a strong risk factor for selection of resistant *Pseudomonas aeruginosa*, and APC restriction has been associated with improvements in susceptibility patterns at the facility level [25,26,27]. Finally, in the absence of a facility penicillin skin testing protocol, implementation of a CDSS-based approach to assessment of β-lactam allergy as adapted from UpToDate may be feasible; although a larger study would be required to determine safety and efficacy.

Strengths of this evaluation include the utilization of interrupted time-series analysis of the primary endpoints, which is the preferred approach for facility-level longitudinal evaluations [28]. The use of APC initiations/1000 PD as an outcome in contrast to the traditional antimicrobial use density measure of DOT/1000 PD is also a strength, as is evaluation of cefepime recipients’ records for findings consistent with β-lactam allergy post-intervention. However, it is important to recognize that we selected advanced generation cephalosporins as a comparator due to probable compatibility with most APC indications; these cephalosporins have a relatively low risk for penicillin cross reactivity as compared to earlier generation cephalosporins based on R side chain structure [29]. Utilization of a CDSS-based approach, which is generally considered to be an effective method for antimicrobial stewardship processes, is also a strength [9,30,31].

**Figure 2:**
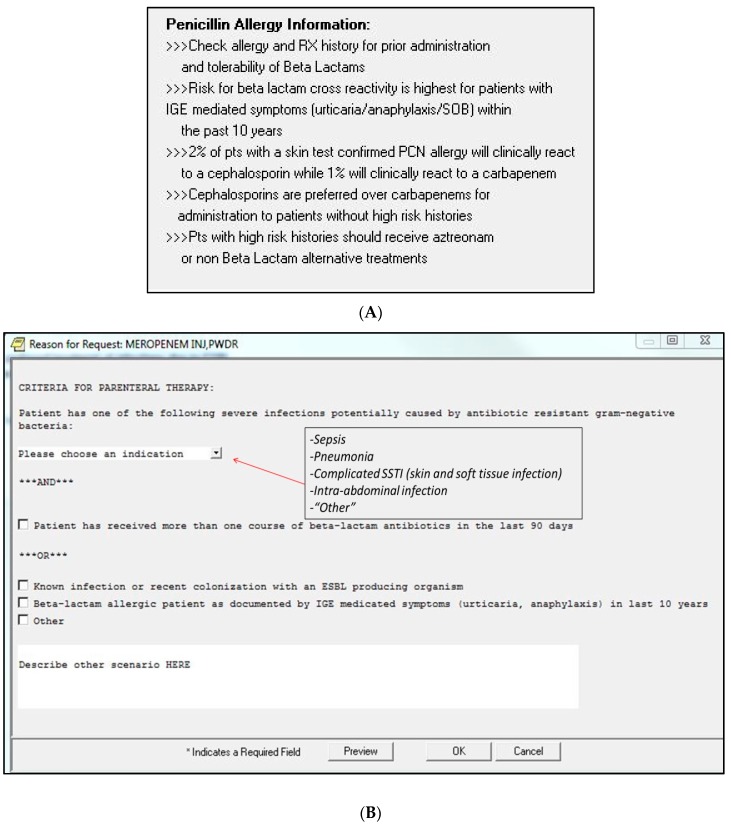
(**A**) Computerized decision support system—information regarding penicillin allergy cross-reactivity with cephalosporins and carbapenems; (**B**) Meropenem order prompt (providers required to complete before order can be submitted).

Limitations include the ecological level of analysis and the single-centered evaluation. Ecological analyses limitations include the potential for regression to the mean, maturation, and the inability to control for important effects [32]. However, the analyses were conducted over 28 months including a 15-month post-intervention period which helped to limit maturation and regression to the mean effects. Another potential limitation is that while we collected data on APC indication, this information was obtained retrospectively from different sources pre and post intervention. Thus, the MUE was not designed to determine the “appropriateness” of APC initiation outside of the context of β-lactam allergy. Further, APC initiation in patients with a history of β-lactam allergy did not decline significantly post intervention. It is unknown if clinicians chose to select alternative antipseudomonal therapy in these cases rather than prescribe an APC, and it is unclear if the sample size was insufficient to detect a difference in the secondary endpoints of APC use in patients with any history of β-lactam allergy. One possibility is that conflicting messages were present within the sequential order menus (Figure 2A,B). Shown in Figure 2A, prescribers were instructed to utilize alternative therapy to an APC in patients at high risk for cross-reactivity; whereas once the decision to prescribe an APC had been made, the menu shown in Figure 2B asked providers to document their choice to prescribe an APC to patients with high risk cross-reactivity. The option to allow clinicians to order a carbapenem and document the “high-risk” criteria in Figure 2B was created with the intent that the potential for an allergic reaction needs to be balanced with appropriate indication for an APC. Pharmacists were instructed to discuss high risk cases with the clinician to confirm their willingness to prescribe a carbapenem and were instructed to contact ASP personnel in questionable cases. Finally, it is possible that patient-level allergy histories were not consistently documented in the electronic medical record, which could have occasionally led to difficulty ascertaining low *vs.* high-risk β-lactam allergy. A literature review reveals that no specific analysis has documented a high prevalence of β-lactam allergy in patients receiving APCs. In larger facilities with ability to perform routine penicillin skin testing, positive impacts on antimicrobial stewardship may be achieved [33]. Although CDSS-based antimicrobial stewardship processes have been reported in literature, many initiatives have focused on utilizing electronic systems to monitor antimicrobial use, or to alert stewardship personnel of positive culture data and/or antimicrobial susceptibility mismatches to enhance the audit and feedback component of stewardship. Our CDSS intervention was a form of criterion-based antimicrobial restriction in that clinicians were required to provide an indication for APC therapy, which has been shown to be an effective tool for reducing broad-spectrum antimicrobial use [9]. CDSS is also increasingly utilized in many small facilities, which can have functionality in assessing antibiotic choice in various infections.

Future work should include analyses to identify the frequency of APC prescription in patients with a history of low-risk β-lactam allergy in different patient populations. If high rates of APC prescribing are identified in patients without indications for APC therapy, further systematic interventions should be conducted to reduce unnecessary carbapenem use in β-lactam allergic patients at low-risk for cross reactivity. Finally, in several cases, the β-lactam allergy was documented as “historical,” with no mention of reaction type or which drug was specifically implicated in the reaction. Improved electronic medical records systems that allow for differentiation of allergy—related symptoms from other medication intolerance and prompt the clinician to enter appropriate information at the time of data entry would be useful.

## 3. Materials and Methods

### 3.1. Design

This was a single-center pre/post intervention retrospective medication utilization evaluation (MUE) was conducted on inpatients who received at least one dose of an APC between 1 July 2012 and 31 October 2014.

At this facility, generally one APC at a time was available based on drug shortages and a formulary change that occurred in 2013. In particular, from 2012 to early 2013, imipenem/cilastatin was the formulary agent; from mid-2013 forward, meropenem was the formulary agent. As these antimicrobials have virtually identical spectra of coverage for most gram-negative organisms, the usage of both agents was combined in this evaluation. Ertapenem, which does not provide coverage against *Pseudomonas* species, was on formulary but restricted and rarely utilized during the period of evaluation. Providers were generally free to select antimicrobials either by directly typing the selected agent into a general medication ordering prompt in the VA Computerized Patient Record System (CPRS), or by selecting an antimicrobial from an inpatient antimicrobial menu that listed formulary and restricted antimicrobials within CPRS.

### 3.2. Intervention

Based on the perception of increased APC use in penicillin allergic patients, a modification to the antimicrobial menu was developed to direct prescribers to appropriately assess a patient’s β-lactam allergy as well as necessity for APC use prior to selection and ordering of antimicrobials. The intervention involved two sequential components. The first antimicrobial menu displayed information about β-lactam cross-reactivity prevalence, and a statement that cephalosporins were preferred over APCs for patients without high-risk β-lactam reactions or need for APC coverage (Figure 2A). Next, if providers selected an APC, a secondary prompt appeared which required the prescriber to indicate whether a patient had a high-risk β-lactam allergy as well as select from a drop-down list an indication for APC use (Figure 2B). Available indication options included sepsis, pneumonia, complicated skin and soft tissue infection, intra-abdominal infection, or “other.” If the provider selected “other,” a text box was provided to allow indication of a diagnosis not available in the drop-down list. Additionally, the provider could indicate that the patient was known to be infected (or colonized for empirical treatment) with an ESBL producing organism, and/or if β-lactam antibiotic use occurred within the past 90 days, both of which were considered as acceptable criteria for treatment with an APC. The antimicrobial ordering menu changes were implemented on 1 August 2013. However, it was observed that providers were not consistently utilizing the inpatient antimicrobial menu but instead were directly typing the APC order into the general medication ordering platform. Thus, on 1 March 2014 the ability to order an APC through the general medication ordering platform was removed, and providers were required to utilize the inpatient antimicrobial ordering menu to order an APC. A conference to house staff on assessment of β-lactam allergy in the context of antimicrobial stewardship was provided in February of 2014.

### 3.3. Data Collection

Patients who received APCs or other antimicrobials of interest were identified through queries of the Veterans Health Information Systems and Technology Architecture and the VA Corporate Data Warehouse. Using a standardized data collection form, patient-level data elements were obtained from CPRS for any patient who received at least one dose of an APC. Patients who received cefepime served as a control for the purposes of comparing adverse events during the post-intervention phase. Data elements collected included age, sex, admitting ward, start/stop date of APC administration, indication for APC therapy, total days of APC therapy, documentation of β-lactam allergy, risk determination of β-lactam cross-reactivity (defined below), and adverse events related to APC or cefepime administration. Indication for APC therapy prescribed before the intervention time-point was determined by reviewing daily inpatient medicine notes. After the intervention, indication for APC therapy was determined by reviewing the electronic APC order, in which the indicated use was specified.

### 3.4. Definitions

High-risk β-lactam allergy was defined as any documented prior history of symptoms consistent with a type 1 IgE-mediated reaction after receiving a β-lactam antibiotic including any of the following: urticaria with wheal and flare, flushing, throat tightness, wheezing, and hypotension (anaphylactic shock) irrespective of the duration of time since the reaction occurred. Low-risk β-lactam allergy was defined as a β-lactam reaction without documentation of symptoms consistent with IgE-mediated hypersensitivity occurring more than 10 years previously, Patients without documentation of the extent of reaction were classified based on the date of allergy observation or entrance into the medical record (*i.e.*, cases that were >10 years ago were classified as low risk).

### 3.5. Outcomes

The primary endpoint was to evaluate whether a change occurred in monthly APC initiations per 1000 patient-days (PD) after the antimicrobial CPRS menu were implemented as compared to the baseline period. The baseline period measured APC use from 1 July 2012 to 31 July 2013, and the post-implementation period evaluated APC use from 1 August 2013 to 31 October 2014. Secondary endpoints included pre/post comparisons of: documented indications for APC use, APC initiations in patients with any history of β-lactam allergy, and APC initiations in patients with low-risk β-lactam allergy. Additionally, documented adverse events for patients with β-lactam allergy who received APCs or cefepime post-intervention were compared.

### 3.6. Statistical Analysis

APC initiations were aggregated by month of study. Interrupted time-series analysis was utilized to determine if the change in antimicrobial menu was associated with a reduction in APC initiations. An indicator variable corresponding to the month that prescribers were required to use the CDSS menu to initiate APC therapy (March 2014) was also tested.

Demographics and other secondary endpoints were tabled and characterized with descriptive statistics and percentages. Fisher’s exact tests were used to compare percentages of documented indications for patients receiving APCs and to compare percentages of APC use in patients with a history of β-lactam allergy pre- and post-intervention. Fisher’s exact test was used to compare percentages of low-risk β-lactam allergic patients pre- and post-intervention.

Analyses were conducted within the Veterans Informatics Computing Infrastructure utilizing R (version 3.1.2, R Foundation for Statistical Computing, Vienna, Austria) and SPSS Statistics (version 21.0.0.0, IBM Corp, Armonk, New York, NY, USA).

This project was reviewed by the institution’s Medication Use Evaluation Committee, a sub-committee of the Pharmacy and Therapeutics Committee. It was determined that this project was an operations activity, not research, and Institutional Review Board approval was not required.

## 4. Conclusions

We observed a high rate of historical β-lactam allergy in patients receiving APCs. A CDSS-based intervention that directed providers to risk-stratify penicillin cross-reactivity potential and assess indications for appropriate carbapenem therapy prior to ordering antimicrobials was associated with a reduction in APC initiations. Similar electronic prompts may be considered for inclusion within CDSSs to facilitate appropriate antimicrobial prescribing in the future.
